# Melatonin Signal Transduction Pathways Require E-Box-Mediated Transcription of *Per1* and *Per2* to Reset the SCN Clock at Dusk

**DOI:** 10.1371/journal.pone.0157824

**Published:** 2016-06-30

**Authors:** Patty C. Kandalepas, Jennifer W. Mitchell, Martha U. Gillette

**Affiliations:** 1 Neuroscience Program, University of Illinois at Urbana-Champaign, Urbana, IL, 61801, United States of America; 2 Department of Cell and Developmental Biology, University of Illinois at Urbana-Champaign, Urbana, IL, 61801, United States of America; University of Texas Southwestern Medical Center, UNITED STATES

## Abstract

Melatonin is released from the pineal gland into the circulatory system at night in the absence of light, acting as “hormone of darkness” to the brain and body. Melatonin also can regulate circadian phasing of the suprachiasmatic nucleus (SCN). During the day-to-night transition, melatonin exposure advances intrinsic SCN neural activity rhythms via the melatonin type-2 (MT_2_) receptor and downstream activation of protein kinase C (PKC). The effects of melatonin on SCN phasing have not been linked to daily changes in the expression of core genes that constitute the molecular framework of the circadian clock. Using real-time RT-PCR, we found that melatonin induces an increase in the expression of two clock genes, *Period 1* (*Per1*) and *Period 2* (*Per2*). This effect occurs at CT 10, when melatonin advances SCN phase, but not at CT 6, when it does not. Using anti-sense oligodeoxynucleotides (α ODNs) to *Per 1* and *Per 2*, as well as to E-box enhancer sequences in the promoters of these genes, we show that their specific induction is necessary for the phase-altering effects of melatonin on SCN neural activity rhythms in the rat. These effects of melatonin on *Per1 and Per2* were mediated by PKC. This is unlike day-active non-photic signals that reset the SCN clock by non-PCK signal transduction mechanisms and by decreasing *Per1* expression. Rather, this finding extends roles for *Per1* and *Per2*, which are critical to photic phase-resetting, to a nonphotic zeitgeber, melatonin, and suggest that the regulation of these clock gene transcripts is required for clock resetting by diverse regulatory cues.

## Introduction

A cyclic pattern of cellular events within the mammalian suprachiasmatic nucleus (SCN) conveys time-of-day information to target organs. Neurochemical inputs relaying information about environmental state, including the pineal hormone melatonin, can modulate the timing of the SCN clock. A high density of melatonin receptors within SCN cells indicates that this locale is a target for melatonin action [1,2,3]. Sensitivity of the SCN to melatonin occurs predominantly during the day-to-night (dusk) and night-to-day (dawn) transitions [1,4]. At these times, melatonin advances the phase of neural activity rhythms generated within the SCN via a protein kinase C (PKC)-dependent pathway that acts downstream of the melatonin type-2 (MT_2_) receptor [5,6,7]. Beyond this, little is understood about the mechanism by which melatonin alters circadian timekeeping.

Light encountered at night is another potent stimulus that shifts intrinsic clock phasing, realigning endogenous rhythms with the cycle of day and night. The resetting action of light on the SCN involves signals from the eye that are transmitted to the SCN where they alter expression of clock genes involved in circadian rhythm generation. In mammals, two genes, *Clock* and *Bmal1*, represent the positive elements of a molecular feedback loop [8,9]. They encode transcription factors that heterodimerize in the cytoplasm and translocate to the nucleus to induce the expression of target genes that contain E-box *cis*-acting regulatory enhancer sequences [9,10]. These include the *Period* (*Per1*, *Per2*, *Per3*) and *Cryptochrome* (*Cry1* and *Cry2*) genes, the negative elements of the circadian feedback loop [11,12], which associate and feedback into the nucleus to repress their own transcription [13]. Activation of *Per1* transcription is a molecular hallmark of the SCN response to nocturnal light, and is necessary for nocturnal light, or glutamate, the key neurotransmitter from the eye to the SCN, to alter SCN phasing [14,15,16]

Using methods that established the relationship between photic signals and their engagement with the SCN circadian clock, we investigated the potential interaction between melatonin and the molecular oscillatory machinery. Using quantitative real-time RT-PCR, we found that melatonin application to the SCN brain slice significantly altered the expression of the clock genes *Per1* and *Per2*, but not *Bmal1*, at CT 10, subjective dusk, the SCN window of sensitivity to effects of melatonin on clock phase. Conversely, the same experiment conducted mid-subjective day, CT 6, when melatonin has no effect on SCN phasing, did not detect changes in *Per1* and *Per2*, although *Bmal1* was reduced. Both the melatonin-induced phase advance of SCN neuronal activity rhythms and regulation of these mRNAs were abolished by specific inhibition of PKC. Moreover, using anti-sense oligodeoxynucleotide (α ODN) decoys, we demonstrate that transcriptional activation of *Per1* and *Per2* via E-box enhancer sequences is necessary for the temporally specific phase shifts induced by melatonin at CT 10. Our findings support a link between melatonin-induced signal transduction pathways activating necessary *Per1* and *Per2* transcription and clock resetting in the SCN by melatonin at dusk.

## Materials and Methods

### Animal Welfare Assurance

All experiments were performed in compliance with the Guide for Care and Use of Laboratory Animals of the National Institutes of Health and approved by the Institutional Animal Care and Use Committee at the University of Illinois at Urbana-Champaign (Animal Welfare Assurance #A3118-01).

### SCN brain slice preparation and circadian time

Long-Evans rats from our inbred colony (LE/BluGill) [17] were maintained on a 12:12 h LD cycle for at least 2 weeks before experimentation. Male animals were between 6–12 weeks of age at the time of use, and received food and water *ad libitum*. For acute brain slice studies, rats were removed from the colony during daytime only (≥ 2 h before the onset of the dark phase) to avoid phase shifts during preparation [18], and quickly decapitated. A 500 μm-thick coronal brain slice containing the paired SCN was prepared and placed at the interface of a Hatton-style brain slice chamber [19]. For qPCR studies, brain slices were reduced in size using a corer (2 mm diameter) to minimize non-SCN tissue, and placed into a brain slice chamber. Slices were perifused continuously with Earle’s balanced salt solution (EBSS) plus 24.6 mM glucose, 26.2 mM sodium bicarbonate, 5 mg/L gentamicin and saturated with 95% O_2_ / 5% CO_2_ at 37°C (final pH 7.2). Slices were under constant illumination and allowed to equilibrate at least 2 h before treatment. Because brain slices are maintained in constant conditions where the clock functions with a period of ~24 h without external time cues, the time of lights-on in the donor colony is designated as circadian time 0 (CT 0). Thus, subjective day (CT 0–12) corresponds to the light portion of the donor’s former lighting schedule, and subjective night (CT 12–24) corresponds to the dark portion of the donor’s cycle.

### Experimental treatments

Melatonin (EMD Chemicals, Gibbstown, NJ) was dissolved at 1 mM in 95% EtOH, and serially diluted to 1 nM in warmed EBSS at the time of use. At the appropriate CT (CT 6 or CT 10), perifusion of the slices was stopped, and a volume of 1 μl containing 1 nM melatonin was applied directly to each SCN for 10 min, after which the SCN was washed with fresh EBSS and perifusion was resumed. Control slices were maintained in parallel in a separate brain slice chamber and treated with 1 μl of EBSS/ SCN for 10 min at CT 6 or 10.

To assess the contribution of PKC to the effect of melatonin, the PKC inhibitor chelerythrine chloride (CC, EMD Chemicals, Gibbstown, NJ) was used. For inhibitor studies, CC was dissolved at 50 mM in 100% dimethyl sulfoxide (DMSO) and diluted to 0.25 mM, 0.05% DMSO in warmed EBSS. CC was bath-applied to the brain slice 20 min before treatment, and then either melatonin or EBSS was applied to the SCN for an additional 10 min. CC at this concentration is sufficient to abolish both melatonin-induced PKC phosphotransferase activity and MT_2_ receptor-mediated phase advances of SCN rhythm in neuronal firing without long-term effects on SCN tissue viability [5,6,20]. For antisense oligodeoxynucleotide (αODN) treatments, slices were pre-treated with αODN for *Per1* or *Per2* for 2 h before the start of melatonin treatment. Perifusion was stopped and the bath media was manually exchanged with medium containing the αODN. To control for non-specific effects of incubation with ODNs, slices were treated in the same manner using a missense ODN sequence.

For E-box ODN treatments, 1 μM of the αODN decoy or missense ODN was bath applied to the brain slice from CT 8–10, and then either melatonin or EBSS was applied at CT 10 for an additional 10 min. At this concentration E-box ODN decoy, but not missense ODN, inhibited the glutamate-induced phase shifts in SCN neuronal activity during early and late night in the rat SCN [21]. At the conclusion of the treatments, the medium in the brain slice chamber was replaced with fresh EBSS, and perifusion was resumed.

### Experimental nucleotide sequence design

Published sequences of αODN against the 5’ start site of *Per1* (5’-TAGGGGACCACTCATGTCT-3’) [14,15,22,23,24] and *Per2* (5’-TATCCATTCATGTCG-3’) [15,22,23] were used (the initiation site is underlined). As a control, corresponding missense ODN also was used (*Per1* missense, 5'-AGCTGCAGCTCTCGGAATT-3’; *Per2* missense, 5’-ACCATGTTACCTGACCTGT-3’) [22,23]. Missense and antisense ODN contain equal GC content. These sequences have been published and successfully used by both our lab [15,25] and others [14,22,23,24] in studies investigating *Per1* and *Per2* involvement in the mammalian clock in rodents.

The E-box decoy was designed and previously used by our lab [25,26]. Briefly, the E-box decoy contains three E-box sequences (5’-CACGTG-3’) plus 6 bp flanking sequence (per E-box) from between exon 1A and exon 1B of the mouse *Per1* promoter (5’- TTTAGCCACGTGACAGTGTAAGCACACGTGGGCCCTCAAGTC CACGTGCAGGGA-3’). The missense control ODN has the same overall nucleotide composition as decoy ODN, but the 5’-CACGTG-3’ E-box sequence has been inverted (5’- TTTAGCGTGCACACAGTGTAAGCAGTGCACGGCCCTCAAGTCGTGCACCAGGGA -3’). Sequences flanking the core E-box remain unaltered in missense ODN.

### Isolation of SCN 2.2 nuclear extracts and electrophoretic mobility shift assay

SCN 2.2 cells were scraped in wash solution (15 mM HEPES, pH 7.2, 250 mM sucrose, 60 mM KCl, 10 mM NaCl, 1 mM EGTA, 5 mM EDTA, 2 mM NaF, 2 mM NaPP_i_, 1 mM phenylmethylsulfonyl fluoride, 5 μM mycrocystin-LR) and centrifuged at 2000 × *g* for 10 min. Pellets were resuspended in cell lysis solution (10 mM HEPES, pH 7.2, 1.5 mM MgCl_2_, 10 mM KCl, 1 mM phenylmethylsulfonyl fluoride 5 μM microcystin-LR, 2 mM NaF, 2 mM NaPP_i_) and centrifuged at 4000 × *g* for 10 min to isolate nuclei. The pellet was re-suspended in nuclei lysis solution (100 mM HEPES, pH 7.2, 1.5 mM MgCl_2_, 1 mM EDTA, 800 mM NaCl, 2 mM NaF, 2 mM NaPP_i_, 1 mM phenylmethylsulfonyl fluoride, 5 μM microcystin-LR, 25% glycerol) and centrifuged at 14,000 × *g* for 30 min to pellet debris. 5 μg of nuclear extract from SCN 2.2 cells transfected with E-box decoy or E-box missense ODN using Effectene (Qiagen) was incubated in 100 ng/ μl poly(dI-dC), 300 nM dithiothreitol, 12 mM Tris pH 8.0, 2 mM MgCl_2_, 60 mM KCl, 120 nM EDTA, pH 8.0, 12.5% glycerol for 30 min at 4°C. 10,000 cpm of ^32^P-end-labeled E-box decoy was added and incubated at 37°C for 10 min. The samples were separated by 8% PAGE and exposed on Kodak MS Film.

### Quantitative real-time RT-PCR (qPCR)

Brain slices were collected at 30 and 120 min following application of melatonin or EBSS (control), and frozen directly on dry ice. Samples were stored at -80°C until use. Isolation of total RNA was performed using TRIzol reagent (Invitrogen, Carlsbad, CA) according to the manufacturer’s protocol. The concentration and purity of the RNA samples was determined by UV spectroscopy.

**Fig 1:**
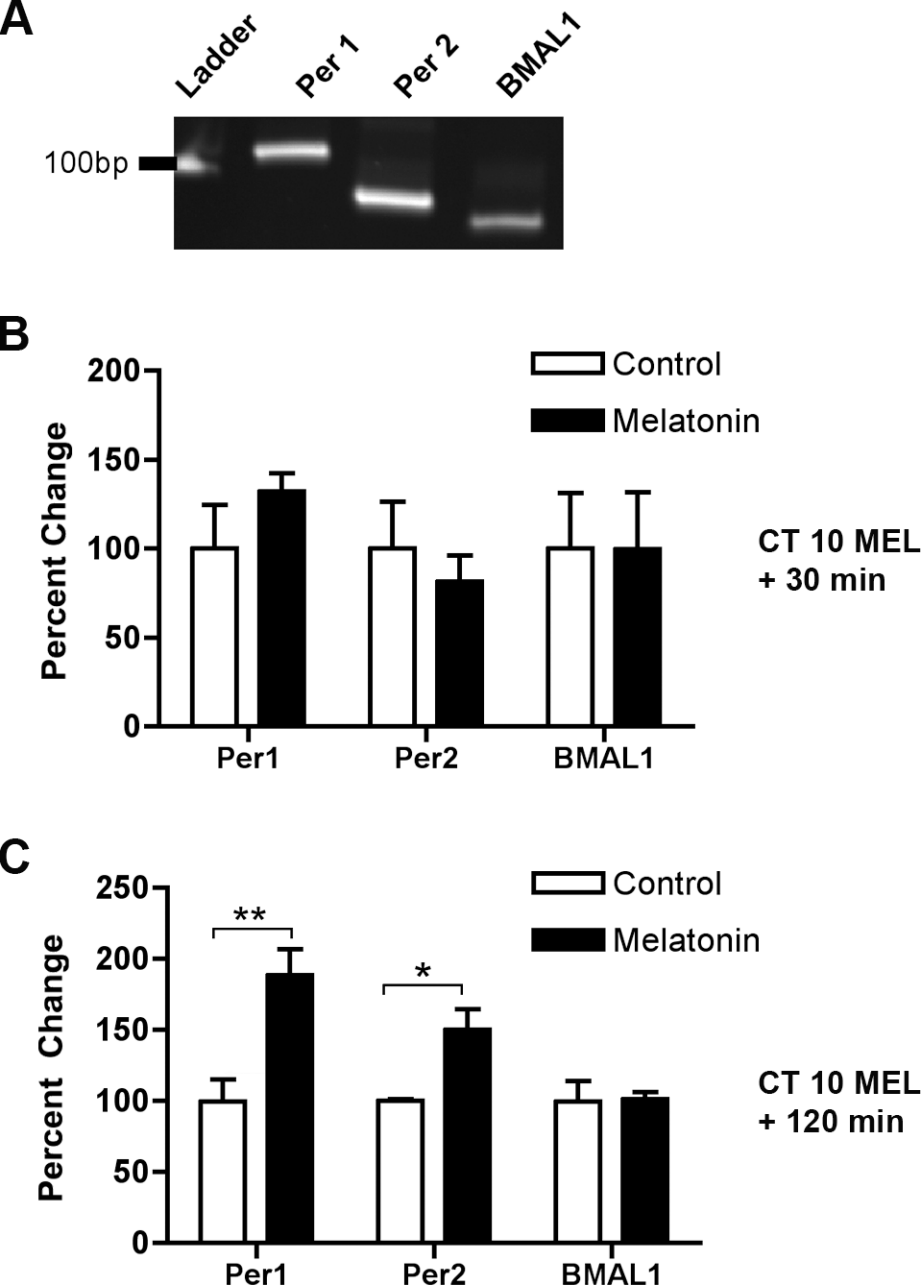
At CT 10, melatonin induces of *Per1* and *Per2* transcription by 120 min. **A)** qPCR amplification products migrate at the predicted size and are distinguishable on an 8% polyacrylamide gel stained with ethidium bromide (*Per1 =* 113 bp, *Per2* = 90 bp, *BMAL1* = 79 bp). **B)** Melatonin has no significant effect on the expression levels of *Per1*, *Per2*, or *Bmal1* mRNA 30 min following the initiation of treatment (p ≥ 0.05, Student’s T Test). **C)** Melatonin treatment significantly increases *Per1* and *Per2*, but not *Bmal1*, transcripts, at 120 min. Data are shown as percent change of relative mRNA levels compared to control ± SEM, *n* = 3-4/condition. ***p ≤ 0.001 (*Per1*), *p ≤ 0.05 (*Per2*), p ≥ 0.05 (*Bmal1*), Student’s T-test.

For the reverse transcription (RT), 0.5 μg of total RNA was annealed to 100 ng random hexamers in a total volume of 12 μl. RT reactions were carried out in duplicate for all experimental samples, and in triplicate for the standard curve. Samples were heat denatured at 75°C for 3 min and quick-chilled on ice to minimize secondary structure present in the RNA. SuperScriptII RNase H Reverse Transcriptase (Invitrogen, Carlsbad, CA) was added in addition to 5x RT Buffer provided with the enzyme, 10 mM DTT, and 0.5 mM dNTP mix to bring the total reaction volume to 20 μl. Reactions were incubated for 1 h at 42°C and heat deactivated for 15 min at 70°C. Samples were diluted to 100 μl with RNase-free water (Invitrogen, Carlsbad, CA) and stored at -80°C until further use. Primer sets were designed to span an intron to avoid amplification from genomic DNA contamination. A BLASTN search was performed against GenBank to evaluate whether the primers were unique to the gene of interest. To avoid amplification from genomic DNA contamination, primer sets were designed to span an intron/exon junction. Sequences are as follows: *rPer1* (Forward: AGGAGGCCCCGGAAGTAGT; Reverse: AGCCTGAAAGTGCATCCTGATT); *rPer2* (Forward: GACGGGTCGAGCAAAGGA; Reverse: GGGAAAAGTCCACATATCC ATTCA); *rBmal1* (Forward: TCTATCCGATGACGAACTGAA; Reverse: CCCTCGGTCACATCCTACAG) (Fig 1A).

All qPCR reactions were carried out in triplicate (BioRad iCycler iQ) with 12.5 μl SYBR Green® Master Mix (Biorad, Hercules, CA), forward and reverse primers (final concentrations 300 nM (*rPer1*), 600 nM (*rPer2*), 300 nM (*Bmal1*)), 5 μl of diluted RT reaction, and RNase-free water to a final reaction volume of 25 μl. The initial denaturation was carried out at 95°C for 10 min, followed by 50 cycles of 50 sec at 95°C and 45 sec at 60°C. The qPCR results are represented as Ct values, or the cycle at which an arbitrary fluorescence threshold was reached. The threshold value was placed such that it was in the exponential portion of the amplification plot for all of the products being generated and was held constant for all runs. A heat disassociation curve was performed at the end of every run to determine that all signals were from an amplicon with the correct disassociation temperature. Negative controls included qPCR conditions that were the same apart from samples that (1) lacked RNA, or (2) lacked the reverse transcriptase enzyme during the RT reaction. Negative controls failed to cross threshold, indicating that the samples were free from amplification from genomic contamination.

A standard curve was generated for each primer set by performing serial dilutions of RNA and plotting the resultant Ct values against the log_2_ of the concentration. The slope, y-intercept, and r^2^ value of the standard curve was determined for each primer set. These values were used to determine the relative mRNA expression level for each experimental sample.

### Electrophysiology

Spontaneous firing rates of individual neurons were assessed *in vitro* using the standard extracellular single-unit recording technique [27,28]. The mean firing rate for individual cells was determined over a 4-min period, and grouped into 2-h running averages with 15-min time lags. Average firing rate was plotted versus the circadian time of recorded activity. The time-of-peak for each experiment was determined from the plot of 2-h running averages for the highest firing rate. Phase shifts were assessed by comparing the mean time-of-peak for treatment groups to vehicle-treated controls. A single peak in spontaneous firing rate of SCN neurons *in vitro* has been described previously [18,29,30,31]. A subset of the recordings were performed with the experimenter blind to the treatment conditions.

### Data analysis and statistics

All qPCR data were plotted as percent change of relative mRNA levels (± SEM) of experimental samples compared to controls (± SEM) that have been set to 100%. Statistical analyses were performed on the averages using a 1- or 2-way analysis of variance (ANOVA), with sample sizes ranging from 3–4 for each treatment. For extracellular recording of SCN neuronal activity, data were plotted as mean firing rate *versus* circadian time of recorded activity. Statistical analyses were performed on the average time-of-peak firing rate of experimental slices *versus* controls using a 1-way ANOVA. Sample sizes were 3–4 for each treatment.

## Results

### *Per1* and *Per2*, but not *Bmal1*, transcripts increase with melatonin at dusk

To test the hypothesis that clock resetting by melatonin is mediated by changes in the expression of core genes involved in circadian rhythm generation, we evaluated levels of *Per1*, *Per2* and *Bmal1* transcripts at CT 10. Assessment of relative levels was made by qPCR. Melatonin application (1 nM, 10 min) to the SCN at subjective dusk (CT 10) had no significant effect on *Per1*, *Per2*, or *Bmal1* mRNA 30 min post-treatment (p ≥ 0.05, *n* = 3, 2-way ANOVA) (Fig 1B). However, melatonin significantly increased *Per1* and *Per2* mRNA compared to controls after 120 min (*p* ≤ 0.001 (*Per1*), *p* ≤ 0.05 (*Per2*), *p* ≥ 0.05 (*Bmal1*), *n* = 3, Student’s T-test) (Fig 1C).

### Melatonin has no effect on *Per1* or *Per2* transcripts during the day, but *Bmal 1* is reduced

**Fig 2:**
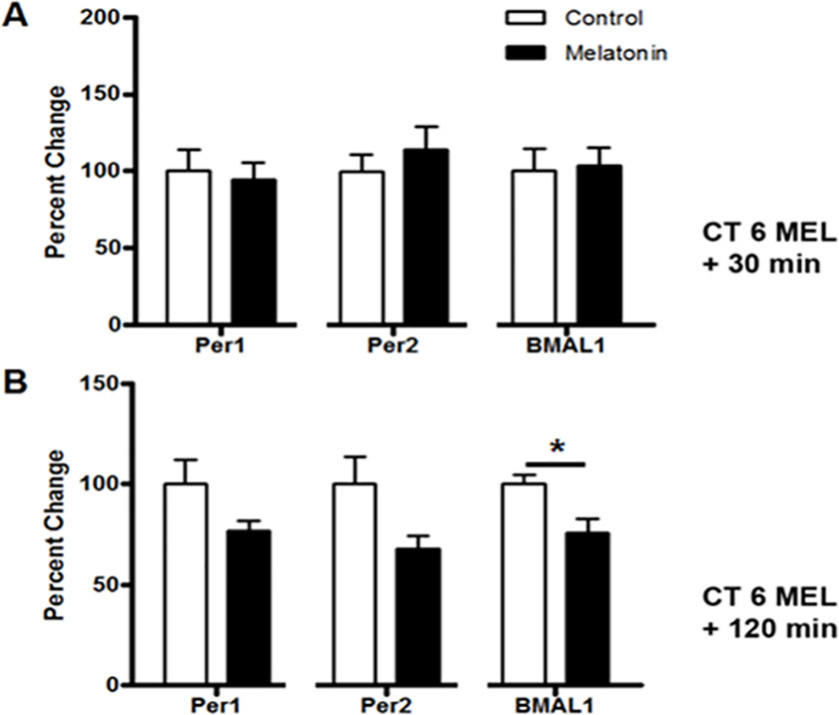
At CT 6, melatonin does not change the levels of *Per1* and *Per2* transcripts, although *Bmal1* is reduced at 120 min. Melatonin applied at CT 6 has no significant effect on the expression levels of *Per 1*, *Per2*, or *Bmal1* mRNA after 30 min **(A)**. After 120 min **(B)**, only *Bmal1* mRNA significantly decreases following initiation of melatonin treatment at CT 6. Data are shown as percent change of relative mRNA levels compared to control ± SEM, *n* = 3–9 /condition, p ≥ 0.05 (*Per 1*, *Per 2*), *p ≤ 0.05 (*Bmal1*), Student’s T-test.

At mid-subjective day (CT 6), when melatonin has no impact on the timing of SCN rhythms in the rat [5], melatonin application had no significant effect on the expression levels of *Per1*, *Per2*, or *Bmal1* mRNA compared to vehicle-treated controls at 30 min or the expression levels of *Per1* or *Per2* mRNA compared to vehicle-treated controls at 120 min following treatment (*p* ≥ 0.05, *n* = 8–9, Student’s T-test) (Fig 2). Only Bmal1 mRNA levels was significantly reduced compared to vehicle-treated controls at 120 min following treatment at CT 6 (p = 0.047).

### PKC activation is necessary for melatonin-induced changes in *Per1* and *Per2* expression at dusk

**Fig 3:**
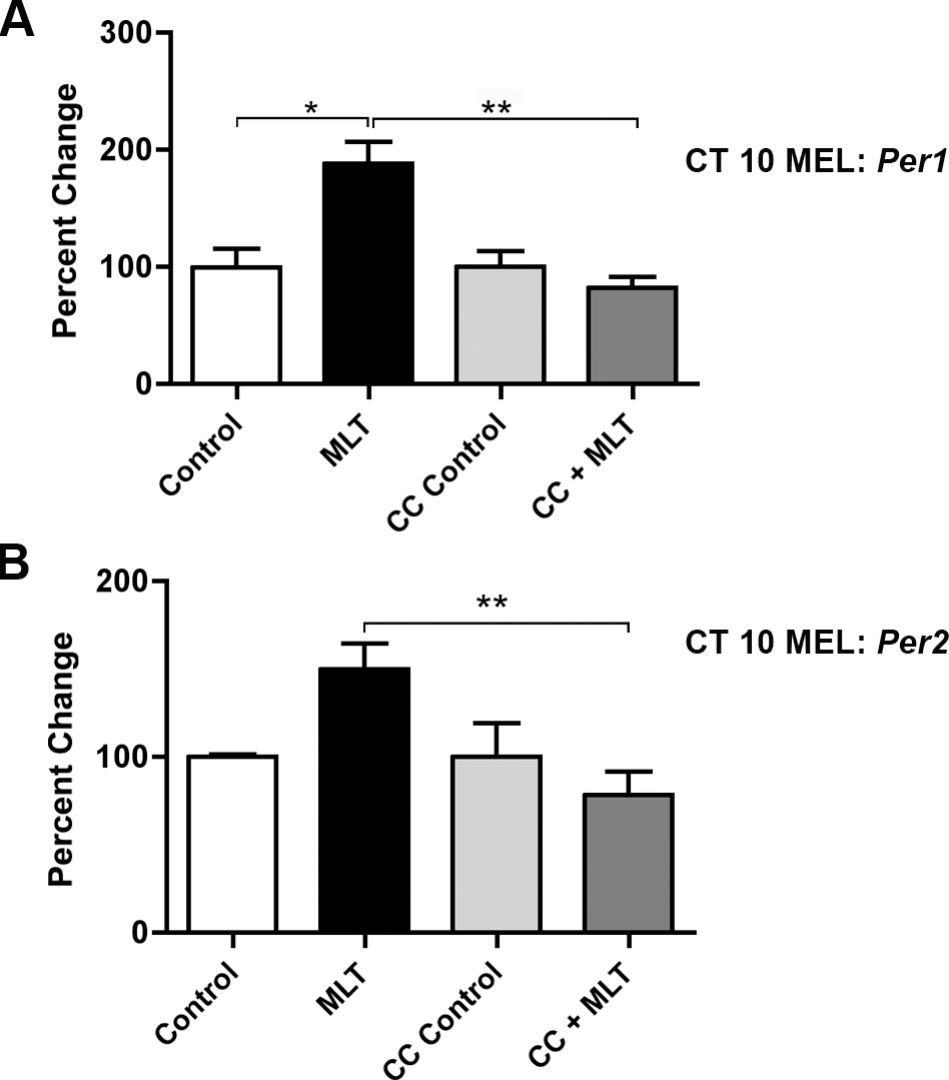
The PKC inhibitor, chelerythrine chloride, blocks the increase of *Per1* and *Per2* mRNA induced by melatonin applied at CT 10. Pre-treatment with 0.25 mM of the PKC inhibitor, chelerythrine chloride, blocks the melatonin-induced increase in *Per1* **(A)** and *Per2* **(B)** transcripts after 120 min. Data are shown as percent change of relative mRNA levels compared to control ± SEM, *n* = 3/condition (** p ≤ 0.01, *p ≤ 0.05, 1-way ANOVA, Tukey’s *post-hoc* analysis). Controls were exposed to sham treatment lacking MEL. MEL = melatonin. CC = chelerythrine chloride.

To test the hypothesis that PKC is a necessary signaling element leading to a change in clock gene expression by melatonin, the PKC inhibitor, chelerythrine chloride (CC), was incubated with SCN slices 20 min before melatonin or EBSS (control) treatment at CT 10. When SCN slices were pre-treated with 0.25 mM CC prior to melatonin, the increase in *Per1* and *Per2* mRNA was abolished (*p* ≤ 0.01 (*Per1*), *p* ≤ 0.01 (*Per2*), 1-way ANOVA) (Fig 3). These results support a role for PKC in the transduction of the melatonin signal to affect clock gene expression in the SCN.

### Increase in *Per1* and *Per2* mRNA is required for melatonin to advance neuronal activity rhythms at CT 10

**Fig 4:**
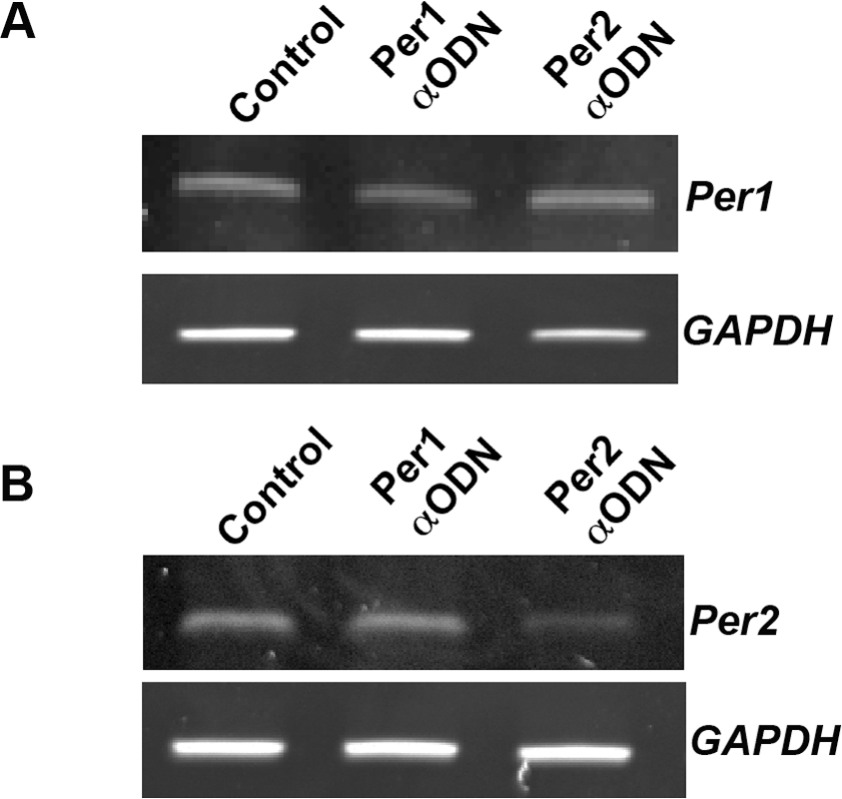
*Per1* and *Per2* αODN attenuate the expression of corresponding transcripts in the SCN. 2-h incubation of SCN slices with αODN results in a 45% decrease in *Per1* transcripts **(A)** and a 60% decrease in *Per2* transcripts **(B)** 4 h after initiation of treatment with the corresponding αODN. No change in GAPDH mRNA was evident following either treatment, which was used as a normalization control.

To determine whether the rise in *Per1* and/or *Per2* mRNA is required for melatonin to induce a phase advance in SCN neuronal activity rhythms, brain slices were pre-treated with αODN targeted to either *Per1* or *Per2* in the absence or presence of melatonin. αODNs act as specific decoys, providing excess, specific binding sites that out-compete native sites for transcription factors. Their use for evaluating the induction of E-box-regulated transcription in the SCN circadian clock is well established [21]. Thus, if E-box-mediated transcriptional activation is required for melatonin-induces phase-shifts, then pre-treatment of SCN slices with an E-box αODN should prevent an increase in *Per1* and *Per2* mRNAs and block a melatonin-induced phase advance in neuronal activity rhythms. A 2-h pulse of αODN (10 μM) from CT 8–10 was sufficient to induce a 45% decrease in *Per1* mRNA, and 60% decrease in *Per2* mRNA, as assayed by RT-PCR (Fig 4). This knockdown is consistent with previous reports using αODN to attenuate *Per* transcripts in the SCN [22,24]. Thus, if melatonin increases *Per1* and *Per2* transcripts to result in a downstream phase-shift, pre-treatment of SCN slices with αODN should prevent an increase in *Per1* and *Per2* mRNAs and block melatonin-induced phase advances in neuronal activity rhythms as previously reported [5,6].

**Fig 5:**
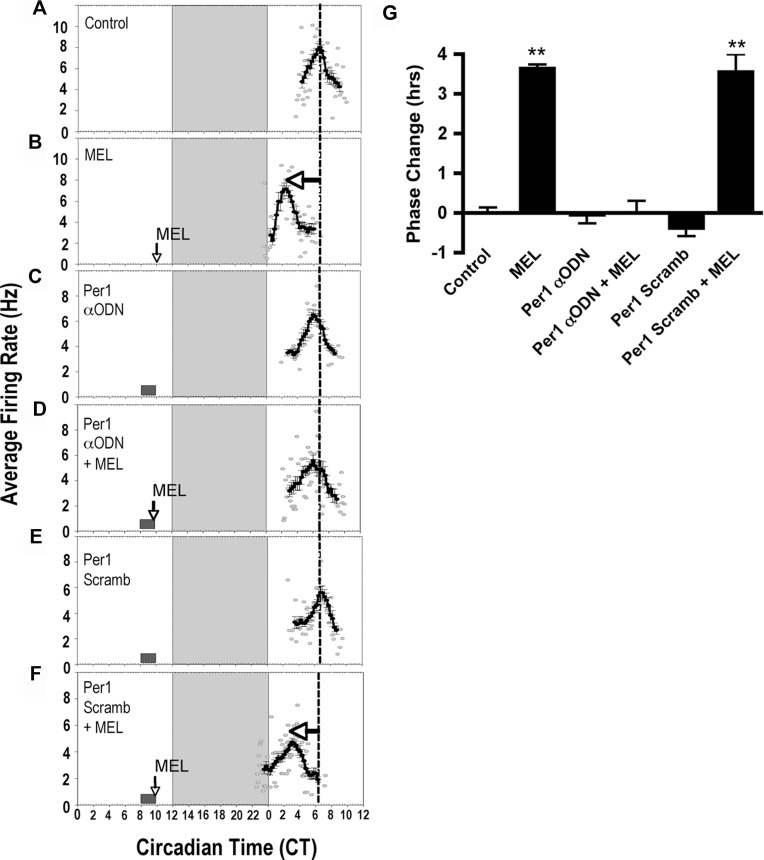
*Per1* is required for melatonin to alter the phase of SCN neuronal activity rhythms at CT 10. **A)** The spontaneous electrical activity rhythm in SCN brain slices peaks at CT 6.38 ± 0.13 in controls. The dotted line indicates the mean time-of-peak for untreated slices. Long, vertical boxes represent subjective night, CT 12–14. **B)** At CT 10, MEL (1 nM, 10 min) advances the electrical activity rhythm by 3.6 h ± 0.10 (*n = 3*). Arrow = time of melatonin treatment. **C)** *Per1* αODN application from CT 8–10 has no significant effect on the time-of-peak electrical activity (*n = 3*). Small box = duration of ODN exposure. **D)** The MEL-induced phase advance is completely blocked by *Per1* αODN (*n = 3*). **E)** *Per1* missense ODN has no effect on the MEL-induced advance in time-of-peak electrical activity (*n = 3*). **F)** *Per1* missense ODN does not block the MEL-induced phase advance at CT 10. **G)** Summary of the effects of *Per1* ODN on MEL-induced phase advances at CT 10. **indicates statistically significant difference compared to controls (p ≤ 0.001) as determined by 1-way ANOVA with Tukey’s *post-hoc* analysis.

The ensemble SCN firing rate in the rat SCN peaked at CT 6.38 ± 0.13 (*n* = 4) in control slices (Fig 5A). Melatonin treatment at CT 10 induced a 3.6-h phase advance in the time-of-peak neuronal activity compared to controls (Fig 5B, mean time-of-peak = CT 2.75 ± 0.10, *n* = 4, *p* ≤ 0.001, ANOVA). *Per1* αODN alone from CT 8–10 had no significant effect on clock timing (Fig 5C, mean time-of-peak = CT 6.42 ± 0.22, *n* = 3, *p* ≥ 0.05, ANOVA), but pre-treatment of the SCN with *Per*1 αODN inhibited the advance in neuronal rhythms induced by melatonin (Fig 5D, mean time-of-peak = CT 6.38 ± 0.30, *n* = 4, *p* ≥ 0.05, ANOVA). To control for non-specific effects of the ODN treatment, brain slices were incubated with *Per1* missense ODN for 2 h before melatonin application. *Per1* missense ODN had no effect alone (Fig 5E, mean time-of-peak = CT 6.75 ± 0.20, *n* = 4, *p* ≥ 0.05, ANOVA) or on melatonin-induced advances in neuronal activity in the SCN (Fig 5F, mean time-of-peak = CT 2.83 ± 0.44, *n* = 3, *p* ≤ 0.001, ANOVA).

**Fig 6:**
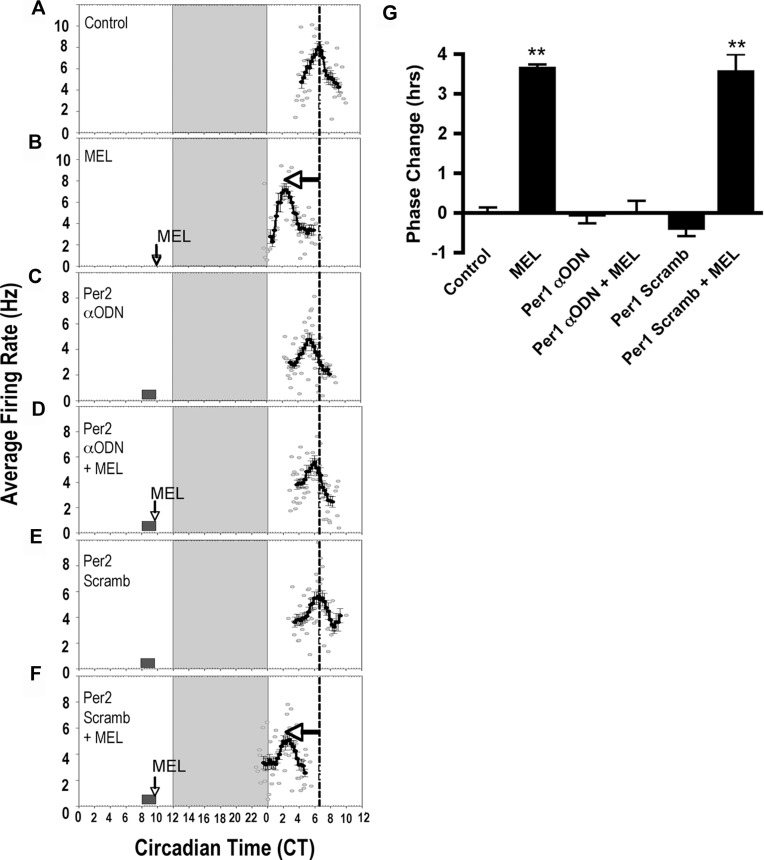
*Per2* is required for melatonin to phase-shift SCN neuronal activity rhythms at CT 10. **A)** The spontaneous electrical activity rhythm in SCN brain slices peaks at CT 6.38 ± 0.13 in control SCN (*n = 3*). **B)** At CT 10, MEL (1 nM, 10 min) advances the electrical activity rhythm by 3.6 h ± 0.10 (*n = 3*). **C)** *Per2* αODN application from CT 8–10 has no effect on the mean time-of-peak electrical activity (*n = 3*). Small box = duration of ODN exposure. **D)** The MEL-induced phase advance is blocked completely by pre-incubation from CT 8–10 with *Per2* αODN (*n = 3*). **E)** Pre-incubation with *Per2* missense ODN has no effect on the MEL-induced advance in time-of-peak electrical activity (*n = 3*). **F)** Pre-incubation with *Per2* missense ODN has no effect on the MEL-induced phase advance at CT 10 (*n = 3*). **G)** Summary of the effects of *Per2* αODN pre-incubations on MEL-induced phase advances at CT 10. **indicates statistically significant differences (p ≤ 0.001) as determined by 1-way ANOVA with Tukey’s *post-hoc* analysis. Symbols as in Fig 5.

Likewise, a 2-h exposure from CT 8–10 to *Per2* αODN alone had no significant effect on SCN clock timing (Fig 6C, mean time-of-peak = CT 6.0 ± 0.23, *n* = 4, *p* ≥ 0.05, ANOVA), but pre-treatment with *Per2* αODN prior to upon melatonin exposure at CT 10 inhibited the phase advance in spontaneous neuronal activity (Fig 6D, mean time-of-peak = CT 6.31 ± 0.12, *n* = 4, *p* ≥ 0.05, ANOVA). Missense *Per2* ODN had no effect alone (Fig 6E, mean time-of-peak = CT 6.5 ± 0.29, *n* = 3, *p* ≥ 0.05, ANOVA) nor on the melatonin-induced phase advance (Fig 6F, mean time-of-peak = CT 2.92 ± 0.17, *n* = 3, *p* ≤ 0.001, ANOVA). These results suggest that melatonin-induced increases at CT 10 in both *Per1* and *Per2* mRNA are necessary to elicit a phase advance in the circadian rhythm of neuronal firing in the rat SCN.

### Decoy ODN blocks binding at E-box sequences in SCN 2.2 cells

**Fig 7:**
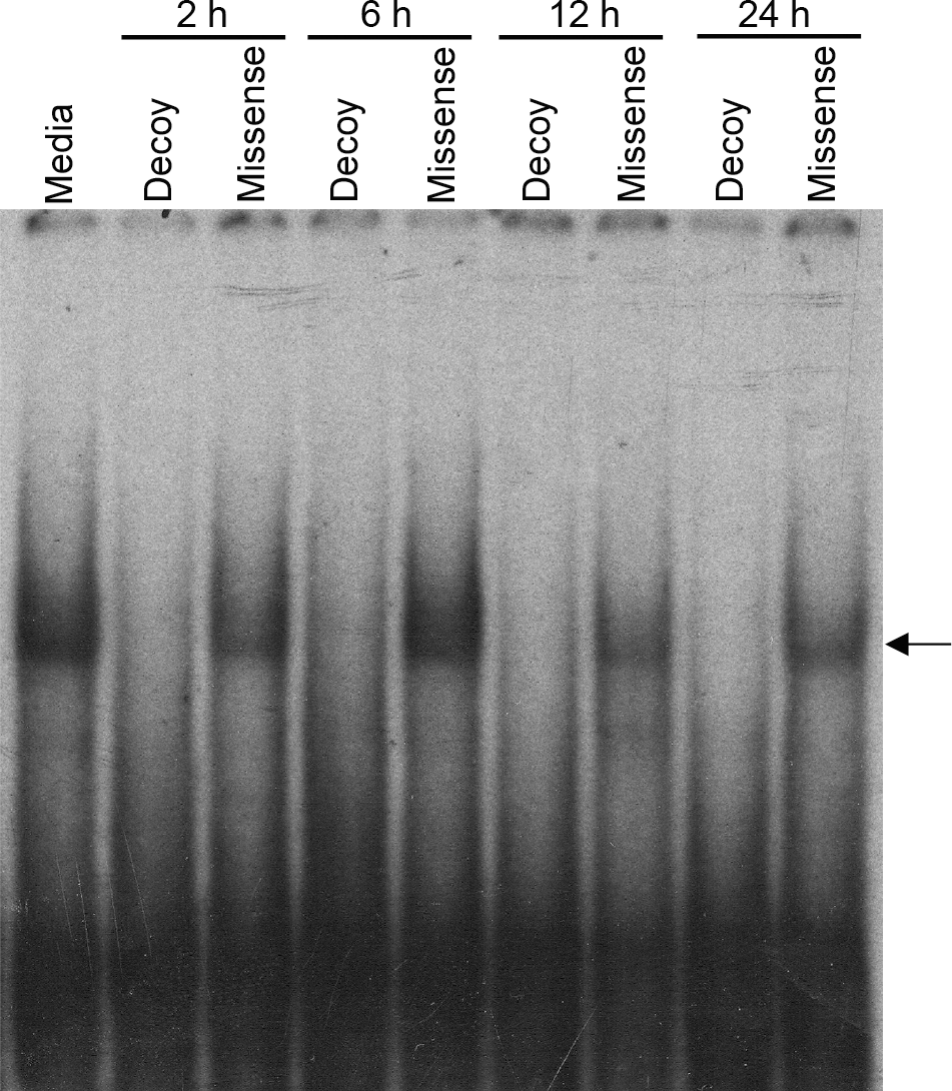
E-box decoy blocks binding at E-box sites in SCN 2.2 cells. Electromobility shift assay of an E-box probe incubated with nuclear extracts of SCN 2.2 cells transfected with 1 μM E-box decoy or missense ODN. Media lane indicates non-transfected control. Arrow = retarded mobility of the E-box probe. This DNA-protein interaction is absent in SCN 2.2 cells transfected with the E-box decoy up to 24 h (*n = 3*).

The E-box decoy is a double-stranded DNA sequence designed to bind to saturate transcription factors that would otherwise bind to E-box sites in the promoters of target genes, thereby preventing their action. To determine whether the E-box decoy could sequester endogenous E-box-binding factors, E-box decoy ODN (1 μM) was transfected into an immortalized SCN cell line (SCN 2.2) and subsequent DNA-protein interactions were assessed by electrophoretic mobility assay. E-box decoy ODN, but not missense ODN, effectively prevented binding of endogenous transcription factors to E-box sites in SCN 2.2 cells for up to 24 h (Fig 7).

### E-box sequences mediate *Per1* and *Per2* expression changes by melatonin

**Fig 8:**
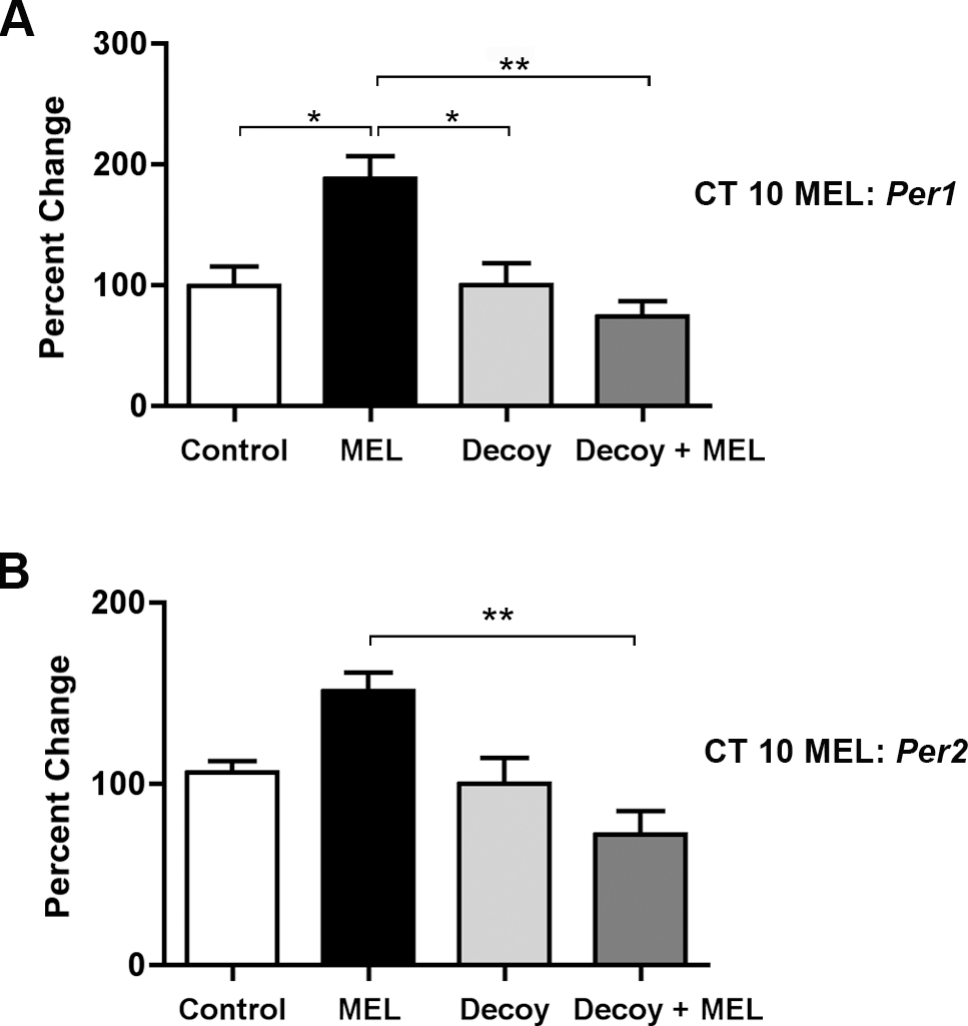
Melatonin-induced increases in *Per1* and *Per2* mRNAs are blocked by E-box decoy ODN. Pre-treatment of SCN slices with E-box decoy ODN (1 μM), blocks the melatonin-induced increase in *Per1* **(A)** and *Per2* **(B)** transcripts after 120 min. qPCR data are shown as percent change of relative mRNA levels compared to control ± SEM, *n* = 3/condition (** p ≤ 0.01, *p ≤ 0.05, 1-way ANOVA, Tukey’s *post-hoc* analysis). MEL = melatonin.

To examine the necessity of the E-box promoter motif for the changes in clock gene expression induced by melatonin, E-box decoy ODN (1 μM) was incubated with SCN slices for 2 h before exposure at CT 10 to melatonin or EBSS (control) treatment. Decoy ODN effectively blocked the increase in *Per1* and *Per2* mRNA at 120 min following melatonin application at CT 10 (*p* ≤ 0.01 (*Per1*), *p* ≤ 0.01 (*Per2*), 1-way ANOVA) (Fig 8).

### E-box-mediated transcription is necessary for melatonin to induce a phase advance in neuronal activity rhythms at CT 10

**Fig 9:**
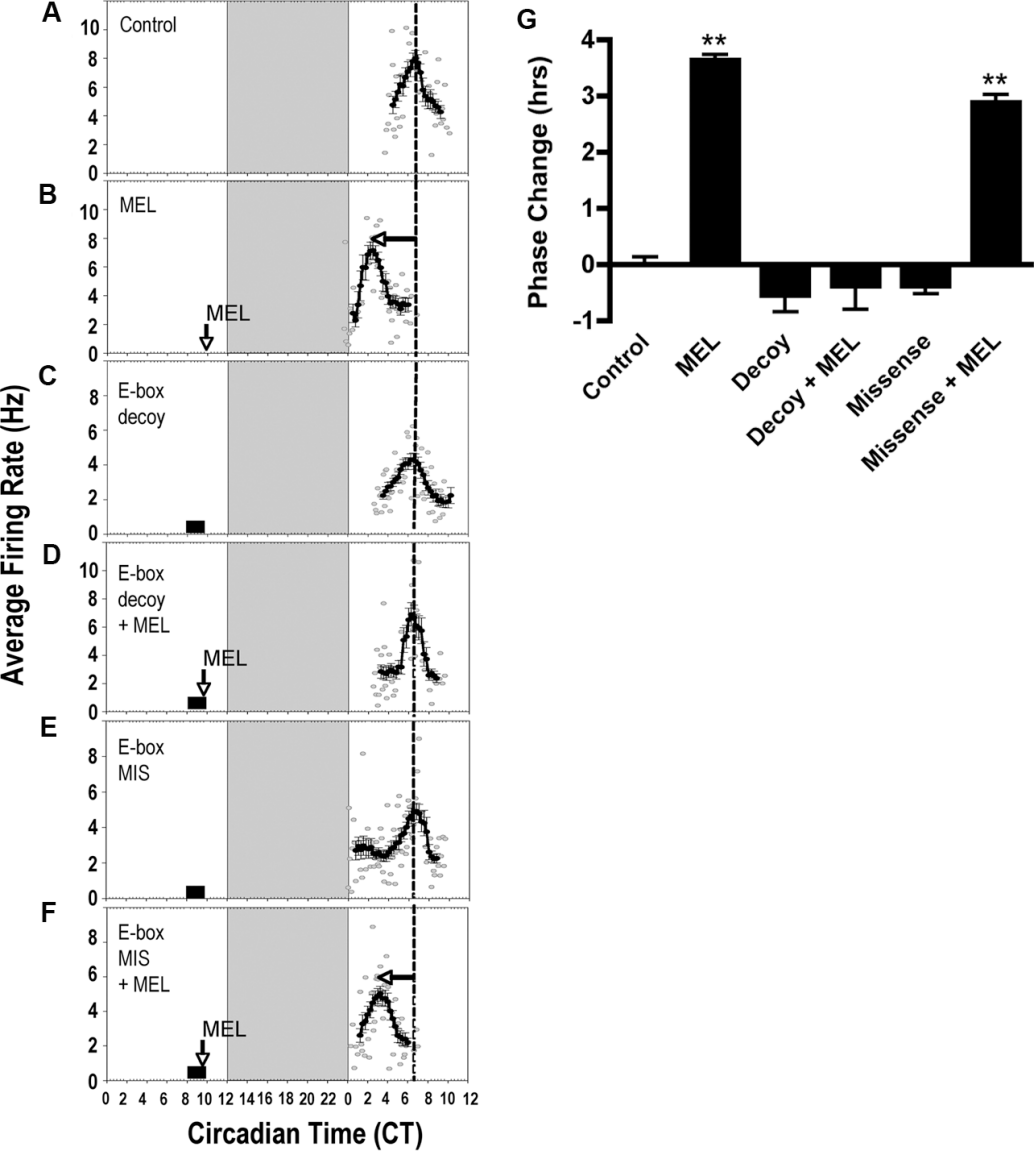
E-box promoter motif is required for melatonin to shift SCN neuronal activity rhythms at CT 10. **A)** The spontaneous electrical activity rhythm in SCN brain slices peaks at CT 6.38 ± 0.13 in controls (*n = 3*). The dotted line indicates the mean time-of-peak for untreated slices. Large, vertical boxes represent subjective night, CT 12–14. **B)** At CT 10, MEL (1 nM, 10 min) advances the electrical activity rhythm by 3.6 h ± 0.10 (*n = 3*). Arrow = time of melatonin treatment. **C)** E-box decoy ODN has no significant effect on the time-of-peak electrical activity (*n = 3*). Small box = duration of ODN exposure. **D)** The MEL-induced phase advance is blocked by the E-box decoy ODN (*n = 3*). **E)** Missense ODN has no effect on the MEL-induced advance in time-of-peak electrical activity (*n = 3*). **F)** Missense ODN does not block the MEL-induced phase advance at CT 10 (*n = 3*). **G)** Summary of the effects of ODN on MEL-induced phase advances at CT 10. **indicates statistically significant difference compared to controls (p ≤ 0.001) as determined by 1-way ANOVA with Tukey’s *post- hoc* analysis.

To assess the role of E-box regulatory elements in the melatonin-induced phase advance in SCN firing rate rhythms, SCN brain slices were pre-treated with E-box decoy or missense ODN from CT 8–10 in the presence or absence of melatonin. The circadian rhythm in SCN firing rate peaked at CT 6.38 ± 0.13 (*n* = 4) in control slices (Fig 9A). Bilateral administration of melatonin to the SCN in brain slices at CT 10 induced a 3.6-h phase advance in the peak of neuronal activity compared to controls (Fig 9B, mean time-of-peak = CT 2.75 ± 0.10, *n* = 4, *p* ≤ 0.001, ANOVA). E-box decoy ODN (1 μM) alone had no significant effect on clock timing (Fig 9C, mean time-of-peak = CT 6.92 ± 0.3, *n* = 3, *p* ≥ 0.05, ANOVA), but abolished the advance in neuronal rhythms induced by melatonin (Fig 9D, mean time-of-peak = CT 6.75 ± 0.42, *n* = 4, *p* ≥ 0.05, ANOVA). After similar pre-treatment with missense ODN alone (CT 8–10), melatonin application at CT 10 had no effect on clock timing (Fig 9E, mean time-of-peak = CT 6.75 ± 0.14, *n* = 3, *p* ≥ 0.05, ANOVA) nor on the melatonin-induced phase advance (Fig 9F, mean time-of-peak = CT 3.5 ± 0.14, *n* = 3, *p* ≤ 0.001, ANOVA).

## Discussion

These studies investigated the hypothesis that melatonin alters SCN clock phasing at dusk via the regulation of core genes that comprise the molecular circadian clock. Melatonin entrains circadian rhythms of wheel-running activity after a sequence of daily injections *in vivo* [32,33,34,35]. Single, brief applications of melatonin to the SCN in a brain slice in the SCN during late subjective day and late subjective night, but not during the day advance the peak of neural activity rhythms [5,36]. Thus, we predicted that melatonin application at CT 10, but not at CT 6, would alter the expression of clock genes. At CT 10, melatonin significantly increased the expression of *Per1* and *Per2*, but not *Bmal1*, an effect that was absent after treatment at CT 6 (Figs 1C and 2). Moreover, phase-resetting effects of melatonin at CT 10 were inhibited by αODN targeted to *Per1* and *Per2*. Changes in *Per1* and *Per2* expression are key for light/glutamate to modulate clock timing during the night [14,23]. These findings extend data on light-induced changes in *Per1* and *Per2* to a nonphotic zeitgeber, melatonin, and suggest that the regulation of these clock gene transcripts is required for clock resetting by diverse regulatory cues.

Light in the early night transiently increases *Per1* transcripts within 30–60 min; levels return to baseline after 3 h [37,38,39]. *Per2* mRNA is induced more slowly, peaking within 2–3 h, followed by a sustained increase for ~6 h [38,39,40]. In the late night, light induces *Per1* mRNA that peaks after 60 min; however, there is no significant effect on *Per2* transcripts at this time [16,39]. The present study demonstrates sensitivity of *Per1* and *Per2* transcriptional activation to melatonin as well, albeit with a slower time course for *Per1* induction by melatonin at CT 10 (Fig 1C). Other nonphotic stimuli, which act in daytime to adjust SCN phasing, induce a decline in *Per 1* and *Per 2* transcripts [41,42,43,44]. Thus, melatonin engages the molecular clock by a mechanism that shares transcriptional targets with light stimulation at night, but is distinct from other nonphotic stimuli.

In the unperturbed SCN, *Per1* levels peak early in the subjective day (CT 4–6), and are relatively low at subjective dusk (CT 10). Non-photic resetting cues that alter clock timing during the day while *Per1* levels are naturally high, induce a decline in *Per1*. However, the induction of *Per1* mRNA by light/glutamate as well as by melatonin suggests that *Per1* is likely to be a gene that is turned on by exposure to a range of phase shifting stimuli at times when *Per1* mRNA is low, from late day through the night. In this case, melatonin is being applied to the SCN at subjective dusk, a time when *Per1* transcript levels are low.

The results described here differ from those reported by Poirel *et al*. [45] showing no effect of melatonin on clock gene expression in the SCN within 24-h of treatment. Differences in experimental design, including methods and conditions of melatonin administration, may account for the differing results. First, Poirel *et al*. [45] used a single subcutaneous injection of melatonin into the rat *in vivo* at CT 11.5; effects of circadian phase were not examined. In contrast, we applied melatonin directly to the SCN in brain slices *in vitro* at CT 10 and demonstrated that this induces phase-resetting of neuronal activity rhythms. Second, previous reports have shown that three consecutive days of single injections *i*.*p*. are necessary to obtain phase-shifting in mice [46]. In humans, four oral doses of melatonin have been used to generate a phase-response curve of the dim-light melatonin onset (DLMO) to melatonin [47]. Additionally, three consecutive days of melatonin administration at the onset of dark in the C3H/HeN mouse facilitated the rate of re-entrainment following an advance in the LD cycle [48]. In a model of seasonal depression, repeated melatonin *i*.*p*. administration at 6 hr into the day of mice exhibiting depressive behavior on a short-day lighting cycle increased the amplitude of clock gene expression, but did not change the phase [49]. Based on the studies cited above, a single melatonin injection *in vivo* is unlikely to have reset the circadian clock and thus unlikely to result in corresponding changes in clock gene expression.

E-box *cis*-regulatory enhancer sequences are DNA elements critical to the molecular feedback loop that underlies circadian rhythmicity, in that they are necessary for transcription initiation of *Per1* and *Per2* [9,50]. In this study, an E-box-specific ODN was used to probe the involvement of E-box promoter elements in melatonin-induced phase resetting. E-box ODN decoys, but not missense ODN, prevented endogenous E-box-binding factors from binding to E-box sequences in the DNA of SCN2.2 cells (Fig 7). This indicates that E-box ODNs can penetrate cells, and can effectively block E-box-specific transcription factors from binding to E-box sequences and activating downstream gene expression. E-box ODN decoys acts as global E-box inhibitors, outcompeting endogenous E-boxes both due to their binding efficiency and by far exceeding the numbers of intrinsic binding sites. In the case of both Per 1 and Per 2, the E-box decoys are competing for the same BMAL/CLOCK dimer pool. Thus, the numbers of available BMAL/CLOCK dimers available to bind to all E-box promoter elements is significantly diminished, and all-E-box-mediated transcription inhibited [25]. Importantly, the effect of the E-box ODN decoy is on the transcription factors themselves, not to the specific E-box sequences.of Per 1 vs. Per 2 promoters.

Pre-treatment of SCN brain slices with E-box decoy, but not missense, ODN effectively blocked melatonin-induced advances in neuronal activity, but had no effect on clock phase when applied to the SCN alone (Fig 9). Similarly, E-box decoy prevented the melatonin-induced changes in *Per1* and *Per2* expression (Fig 8). These results implicate the E-box promoter element in generating shifts in the SCN clock by melatonin at subjective dusk. We are unaware of prior evidence linking the E-box promoter motif to the melatonin-induced phase advance in SCN neuronal activity rhythm. However, we identified the E-box element as a necessary target for clock resetting in the glutamate signaling pathway in the SCN [26]. This suggest that melatonin and glutamate signals both target activation of *Period* gene expression via binding of transcription factors to E-boxes for circadian clock resetting.

There are several mechanisms by which melatonin may affect E-box-dependent transcription in the SCN. The melatonin signal may positively regulate gene transcription by facilitating the binding of CLOCK/BMAL1 heterodimers to E-box containing promoters and recruiting transcription factors [9,51]. Alternatively, melatonin may activate already-bound CLOCK/BMAL1 complexes. This could occur via recruitment of co-activators, which may trigger the intrinsic histone acetyltransferase (HAT) activity of CLOCK [52], thereby making the DNA more accessible to transcriptional machinery. Either scenario would promote the initiation of *Per1* and *Per2* transcription.

Advances in our understanding of the molecular clockwork mechanism include a role for post-translational modifications affecting clock protein function [53]. This raises the possibility that the melatonin signal may influence the transactivation potential of DNA binding proteins via protein kinases. There is evidence that mammalian CLOCK is phosphorylated and activated via a Ca^2+^-dependent PKC pathway [54].

Alternatively, melatonin signaling components may affect *Per1* and *Per2* expression by relieving transcriptional inhibition at E-box promoters. There is evidence for this type of regulation, whereby the DEC (differentially expressed in chondrocytes) transcription factors, DEC1 and DEC2, repress CLOCK/BMAL1-induced transcriptional activation of the mouse *Per1* gene [55]. Perhaps melatonin facilitates the de-repression of CLOCK/BMAL1 at the level of DEC1 and/or DEC2, thereby inducing *Per1* and *Per2* gene expression. However, there is currently no evidence linking melatonin and activation of DEC proteins.

We have examined the effect of the E-box decoy ODN alone and in combination with melatonin in the rat hypothalamic brain slice *in vitro*, and conclude that the E-box promoter motif is required for transmitting signals of change by melatonin to the circadian clock at CT 10 *in vitro*. The contribution of CLOCK/BMAL1-induced activation of *Per1* and *Per2* expression to alter clock timing by melatonin is unclear at this time, but warrants further investigation. These data support the notion that the regulation of clock genes by upstream activating sequences is required for clock resetting not only by light, but also by other regulatory stimuli, including the pineal hormone melatonin.

Melatonin alters clock phase by activating PKC [5]. A specific inhibitor of PKC, chelerythrine chloride (CC), blocked the changes induced by melatonin in *Per1* and *Per2* transcripts (Fig 3). These findings place melatonin, MT_2_ receptor activation, PKC activation, and downstream induction of *Per1* and *Per2* mRNA via E-boxes in a signaling cascade mediating phase advances at CT 10. We previously have shown that melatonin also alters clock phasing at CT22, subjective dawn in the SCN. As during subjective dusk at CT 10, PKC signaling is necessary for the effect of melatonin on SCN phase at dawn [5]. The effect of melatonin on clock genes at this important circadian time warrants further investigation.

Investigations into the molecular nature of the mammalian timing system have defined a general framework for central clock mechanisms, although understanding is still incomplete. The mammalian circadian clock is extraordinarily complex dynamic system, responding to a diverse and growing list of phase-shifting stimuli. Each stimulus potentially encodes a distinct time-of-day signature that adjusts the SCN oscillatory machinery so as to synchronize with day and night. Specific time-of-day effects of melatonin on *Per1* and *Per2* transcriptional activation that wax and wane are consistent with the dynamic nature of the timekeeping mechanism. The changes in *Per1* and *Per 2* expression induced by melatonin, a non-photic stimulus, during the approach tonight support a transcriptional induction of change that shares some components with mediators of effects of light on the SCN clock several hours later, during the night.
